# A combination of approaches evidenced seed storage behaviour in the miracle berry *Synsepalum dulcificum* (Schumach. et Thonn.) Daniell

**DOI:** 10.1186/s12870-019-1714-1

**Published:** 2019-03-29

**Authors:** Dèdéou Apocalypse Tchokponhoué, Sognigbé N’Danikou, Enoch Gbènato Achigan-Dako

**Affiliations:** Laboratory of Genetics, Horticulture and Seed Science (GBioS), School of Plant Sciences, Faculty of Agronomic Sciences, University of Abomey-Calavi, Abomey-Calavi, Republic of Benin

**Keywords:** *Richardella dulcifica*, Recalcitrance, Seedling growth, Sustainable conservation

## Abstract

**Background:**

Knowledge on seed storage behaviour is crucial for planning conservation strategies of plant genetic resources particularly in economically promising but endangered species like *Synsepalum dulcificum*, viewed as recalcitrant-seeded species albeit sound evidence was lacking. In this study, we combined an experimental approach based on critical moisture content and storage environment analysis, and the seed-coat ratio–seed dry mass (SCR-SM) model to clarify the seed storage behaviour in the species. Seed moisture content at shedding was determined and effects of dehydration and cold storage on seed viability, germination and subsequent seedling vigour were analysed. The probability for dessication-senstivity [P(D-S)] was also determined.

**Results:**

Our findings indicated that *S. dulcificum* seed moisture content at shedding was 36.60% with nearly 100% viability. Seed dehydration below 20% moisture content induced a total loss of viability whereas low temperature storage (at 10 °C or 4 °C reduced shelf life to a maximum of 7 days. More importantly, *S. dulcificum* seed storage at 0 °C was highly detrimental and resulted in a total loss of viability whatever the storage duration. Only a storage at 25 °C helped expand the shelf life to 28 days. However, at 28 days storage the viability was extremely low with almost no germination. The probability for dessication-senstivity P(D-S) in the species is largely greater than 0.5. Seed dehydration and storage environment highly affected subsequent germination rate and seedling vigour. While dehydration improved seedling performance storage at low temperature rather inhibited seedling growth.

**Conclusion:**

Taken together, these findings are the first to set evidence of recalcitrance in *S. dulcificum* and serve hands-on information for practical handling of the seeds and designing sustainable conservation practices for adequate future breeding programme in the species.

**Electronic supplementary material:**

The online version of this article (10.1186/s12870-019-1714-1) contains supplementary material, which is available to authorized users.

## Background

Knowledge on seed storage behaviour is crucial for planning conservation strategies of plant genetic resources [1]. Plant species can be classified into three groups based on the desiccation level and the storage temperature their seeds tolerate: orthodox-seeded, intermediate-seeded and recalcitrant-seeded species [2, 3]. Orthodox seeds are likely to tolerate severe desiccation (below 0.05 g g^− 1^ of water content) and can be stored at sub-zero temperatures without damage [4]. They have a wide hydration window [5]. Difficult-to-store seeds are categorized as intermediate and recalcitrant types. Recalcitrant seeds are very sensitive to desiccation [6] and do not survive drying to relative low moisture content [2]. The intermediate species are sensitive to both low moisture content and low storage temperature combined with a very narrow hydration window [7, 8]. Due to that high sensitivity of recalcitrant species, their seeds are viable only for a very limited period and exhibit an inability to medium and long term storage [3]. Conservation of recalcitrant and intermediate-seeded species is considered as one of the most challenging issues seed scientists are facing, reducing ex-situ options [9]. In this twenty-first century characterized by climactic variability accurate information on the seed storage behaviour is needed for defining propagation and conservation strategies.

It is known that more than 25% of worldwide plant species produce recalcitrant species [9] and that more than 8% of world’ flowering plant produce difficult-to-store seeds [10] out of which many exhibit huge economic and food values (e.g. *Artocarpus heterophyllus* Lamk., *Coffea* spp., and *Theobroma cacao* L.). For a long time, classification of plant species into one or the other of the three seed storage behaviour (SSB) groups had been a challenge. Indeed, the first list of recalcitrant-seeded species was revised two times and shortened firstly from 73 to 68 species and secondly from 68 to 49 species [11, 12]. Later, Hofmann and Steiner [13] proposed another list of 186 recalcitrant species excluding 19 species of the former list (e.g. *Elaeis guineensis* L. and *Azadirachta indica* L.). Afterwards, 17 species (e.g. *Flacourtia indica* (Burm.f.) Merr*., Fagraea fragrans* Roxb.) initially classified as orthodox-seeded by Hong et al. [14] were reclassified as recalcitrant-seeded by Farnsworth [15]. Nevertheless, advances in the field of seed science during the last two decades have resulted in the use of more accurate but simplified approaches or even combined approaches to overcome challenges in the determination of seed storage behaviour. For instances, Oliver et al. [16] used metabolomics approach to highlight difference in seed storage behaviour of two sister species (*Sporobolus pyramidalis* P.Beauv. and *S. stapfianus* Gand.) whereas Delahaie et al. [17] used proteomics approach to illustrate difference in seed storage behaviour of *Castanospermum austral* A. Cunn. & C. Fraser and *Medicago truncuntala* Gaertn. Similarly, Pritchard et al. [18] and Daws et al. [19] respectively proposed 100-seed test and Seed-coat ratio–seed dry mass (SCR-SM) models for rapid seed storage behaviour assessment, particularly in woody species. An ongoing trend in SSB allocation has been the combination of several approaches resulting in more reliable conclusion [20, 21] and appeared relevant for the rapid and reliable study of the nearly 311,000 plant species for which information on seed storage physiology is still needed [18].

In the case of *Synsepalum dulcificum* (Schumach. et Thonn.) Daniell [syn: *Richardella dulcifica* (Schumach et Thonn.) Baehni], a poorly documented species, some authors suggested that the species was recalcitrant-seeded, likely because seeds rapidly lose viability when air-dried [22, 23]. However, Hong and Ellis [3] have showed that rapid seed viability loss was not specific to recalcitrant-seeded species. For instance, in the genus *Salix*, reported as dominated by orthodox-seeded species, seeds lose viability within 2 days in open storage [24].

*Synsepalum dulcificum* is an African native shrub species with socio-economic values and great potentials in food, pharmaceutical and cosmetic industries. The species is worldwide recognized for its “miraculin” a glycoprotein with sweetening activity and represents a substitute to synthetic sugar for sour beverages [25]. Detailed information on utilizations and potentials of the species are presented in Achigan-Dako et al. [26]. With the increasing awareness of the use of the species to reduce diabetes in the world, basic knowledge is becoming crucial to define crop improvement schemes. To that end, a proper handling of germplasm and seed batches will facilitate management of genetic resources of the species. Although the species belongs to the Sapotaceae, a plant family ranked third in term of proportion of recalcitrant-seeded species (in 64.5% of the sampled species within the family) [27], the seed storage behaviour in the genus *Synsepalum* and especially in *S. dulcificum* was never properly elucidated. In addition, the conclusion above was based on 2.4% only of worldwide Sapotaceae species. Moreover, Hong and Ellis [3] suspected intermediate-seeded species in the family indicating that there is a need to carry out species-specific investigations so as to have a clear behaviour delimitation. Furthermore, the Seed Information Database [28] indicated *S. dulcificum* as “likely recalcitrant”, calling for the necessity for further investigation to ascertain the species seed storage behaviour.

The aim of this paper is therefore to use a proof-driven approach to clarify the seed storage behaviour of *Synsepalum dulcificum*. We hypothesized that i) *Synsepalum dulcificum* seeds are truly recalcitrant, and ii) dehydration and storage decrease both seed vigour and seedling growth in *S. dulcificum*. We combined experimental and theoretical approaches to analyse how dehydration and storage affect the seed vigour and subsequent early seedling growth.

## Results

### Association between fruit colour and germination performance in *Synsepalum dulcificum*

**Fig. 1:**
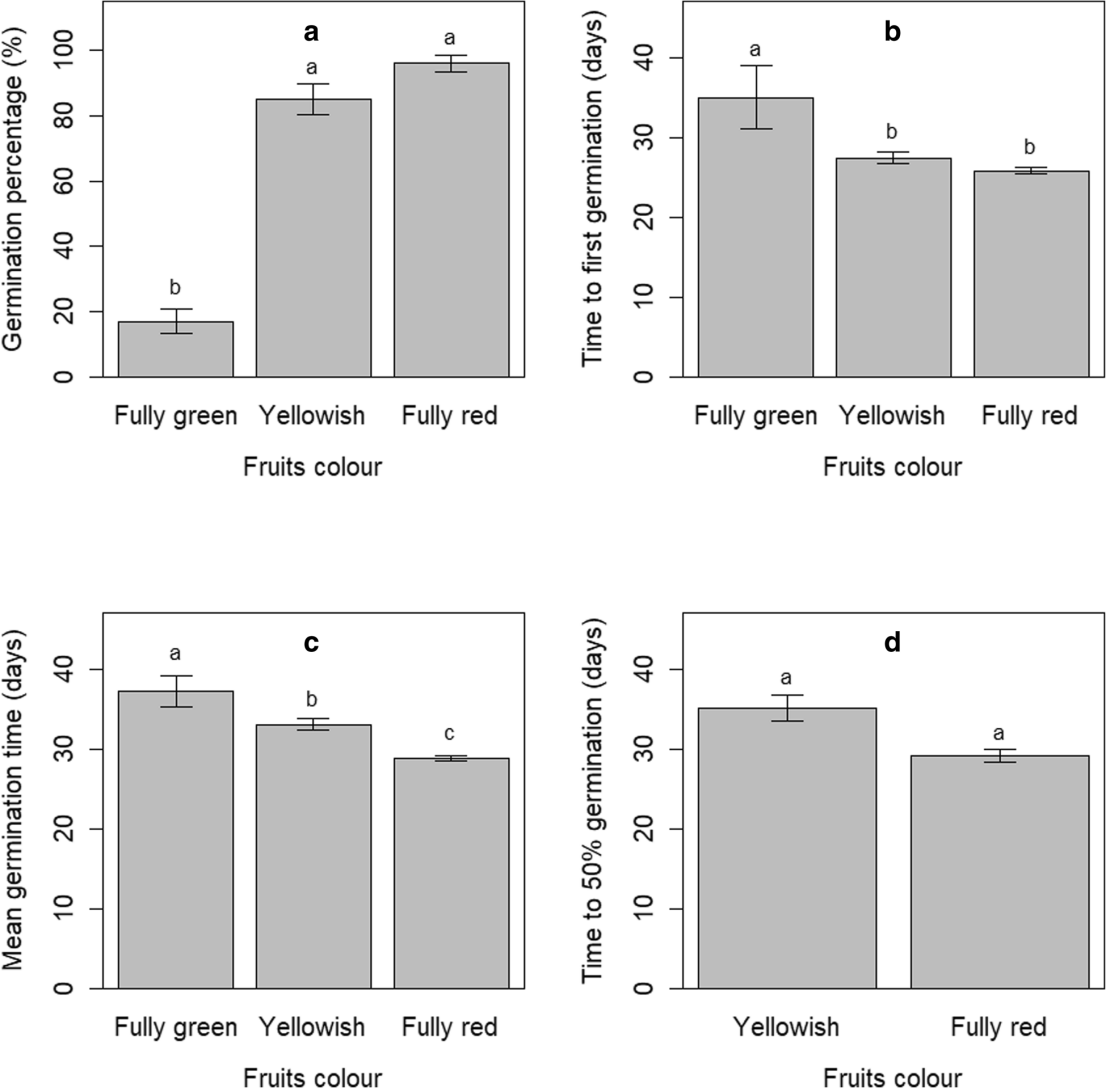
Effect of fruit colour of the germination performance of *Synsepalum dulcificum*. (**a**) Germination percentage. (**b**) Time to first germination. (**c**) Mean germination time. (**d**) Time to 50% germination. Error bars indicate standard error (*n* = 5 replicates, each replicate having 20 seeds). Different lowercase letters denote statistical differences among treatment groups according to Tukey HSD test

Germination percentage greatly differed among fruits of different colours (*P* <  0.001) (Fig. 1A). Yellowish and fully red fruits had higher germination percentages whereas green fruits exhibited a lower germination percentage. Likewise, the time to the first germination was lower in yellowish and red fruits than in green fruits (*P* <  0.01) (Fig. 1B) whereas, overall, fully red fruits germinated quicker than any other fruits (*P* <  0.001) (Fig. 1C). While green fruit did not reach 50% germination, yellowish and red fruits did so, in a relatively similar timeframe (*P* > 0.05) (Fig. 1D). For all evaluated parameters, the best performance was constantly observed in fully red fruits. These indicated that red fruits of *S. dulcificum* ensured sufficiently mature seeds to serve in unravelling the seed storage behaviour in the species. For subsequent experiments, we exclusively used seeds from the fully red fruits.

### Seed storage behaviour in *Synsepalum dulcificum*

The fresh mass of 100 seeds in *S. dulcificum* was 19.44 ± 0.18 g. The seed moisture content (m.c.) at shedding was 36.6 ± 0.79%.

**Fig. 2:**
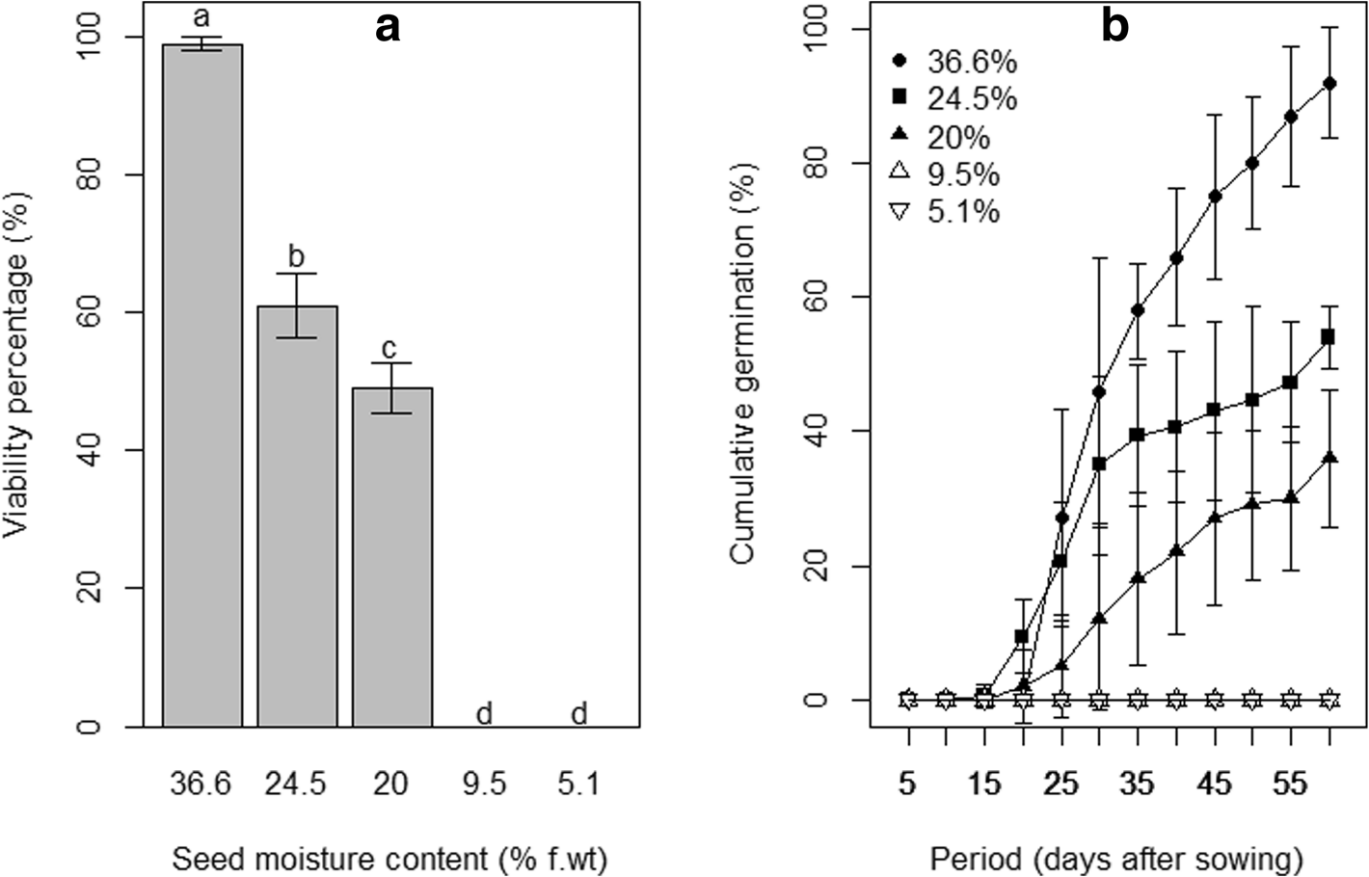
Effect of dehydration on seed viability (**a**) and seed germination (**b**) in *S. dulcificum*. Error bars indicate standard error (n = 5 replicates, each replicate having 20 seeds). Different lowercase letters denote statistical differences among treatment groups according to Tukey HSD test

At shedding seed viability was 99 ± 1%. This viability was significantly reduced by dehydration (*P* <  0.0001). It decreased from 99% in fresh seeds (36.6% m.c.) to 49% (20% m.c.) and 0% below 10% moisture content (Fig. 2A, Additional file 1). Similarly, dehydration negatively affected germination percentage (*P* <  0.0001) (Fig. 2B). No germination was observed in seed dehydrated to 9.5 and 5.1% m.c., whereas germination percentage decreased by nearly two third when seed moisture content is reduced by half (Fig. 2B, Additional file 1). Only fresh seeds and seeds dehydrated to 24.5% reached 50% germination.

**Fig. 3:**
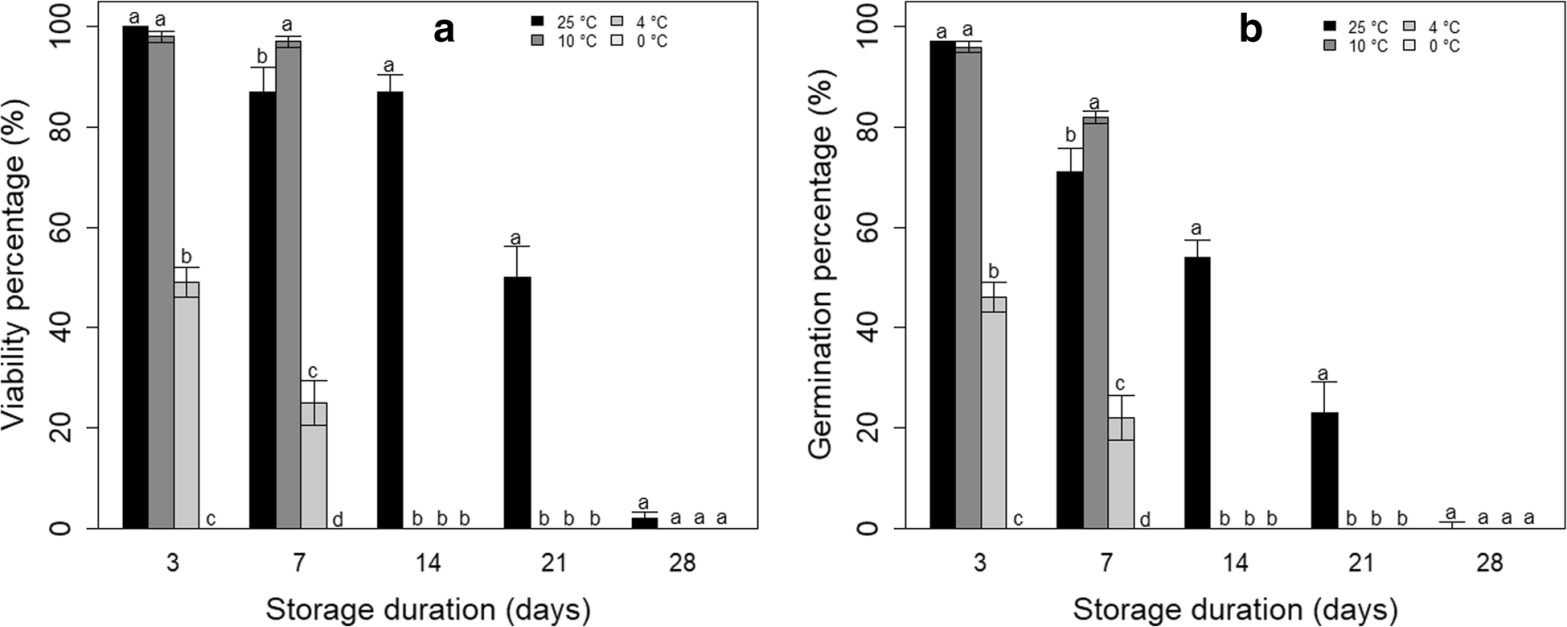
Viability (**a**) and germination (**b**) percentage of seeds (initial moisture content: 36.6%) of *S. dulcificum* based on storage duration and temperature. Error bars indicate standard error (n = 5 replicates, each replicate having 20 seeds). Different lowercase letters denote statistical differences among treatment groups according to Tukey HSD test

Across the experimental storage, viability in *S. dulcificum* ranged from 0 to 100%. There was a highly significant (*P* <  0.0001) interaction effect between the temperature of storage environment and the storage duration on seed viability and germination. Seed viability and germination decreased with a decrease of storage temperature and with increased storage duration (Fig. 3A and B, Additional file 2). Seed storage at 0 °C was highly detrimental for *Synsepalum dulcificum* whatever the storage duration. Data also showed that storage at low temperature (4 °C) reduces seed viability to half within 7 days, while half of seeds stored at temperature close to ambient conditions (25 °C) still conserve their viability at 21 days storage. Viability is lost in all seed lots after 7 days for seeds stored at 4 °C/10 °C whereas from 28 days storage for those stored at 25 °C, the viability and the germination were almost nil. Though more abrupt, the trend in the germination decrease was comparable to that of viability decrease (Fig. 3B). The highest germination percentages were obtained in seed stored for a maximum of a week either at 25 °C or at 10 °C.

The probability for desiccation-sensitivity [P(D-S)**]** derived from the seed-coat ratio–seed dry mass (SCR-SM) model ranged from 0.76 to 0.87 (Table 1). Though the P(D-S) was slightly higher in seeds from juveniles (3 years old and 45–50 cm tall tree), neither the seed origin nor the mother plant growth stage affected the trend in the probability for desiccation-sensitivity (*P* > 0.05).

**Table 1:** Probability for desiccation-sensitivity [P(D-S)] range in *Synsepalum dulcificum*

Seed origin	Mother plant growth stage	Individual	SCR	Log_10_ (seed mass)	P(D-S)
Abomey-Calavi	Juvenile^a^	Juvenile 1	0.210	0.372	0.87 ^a^
Abomey-Calavi	Juvenile^a^	Juvenile 2	0.239	0.44	0.86 ^a^
Abomey-Calavi	Juvenile^a^	Juvenile 3	0.210	0.23	0.84 ^a^
Klouekanmey	Adult^b^	Adult 1	0.210	0.107	0.8 ^a^
Sèhouè	Adult^b^	Adult 2	0.240	0.227	0.79 ^a^
Sey	Adult^b^	Adult 3	0.247	0.168	0.76 ^a^
*P* = 0.34^ns^	*P* = 0.26^ns^	*P* = 0.27^ns^			

*SCR* Seed-coat ratio. Values within columns followed by the same letter are not significantly different (Binomial test). ^a^Three years old and 45–50 cm tall trees; ^b^Sixty years old trees with more than three meters height

### Effect of dehydration and storage temperature and duration on germination rate in *Synsepalum dulcificum*

Germination rate was measured using three parameters: mean germination time (MGT), time to first germination (TFG) and time to 50% germination (T_50_).

**Fig. 4:**
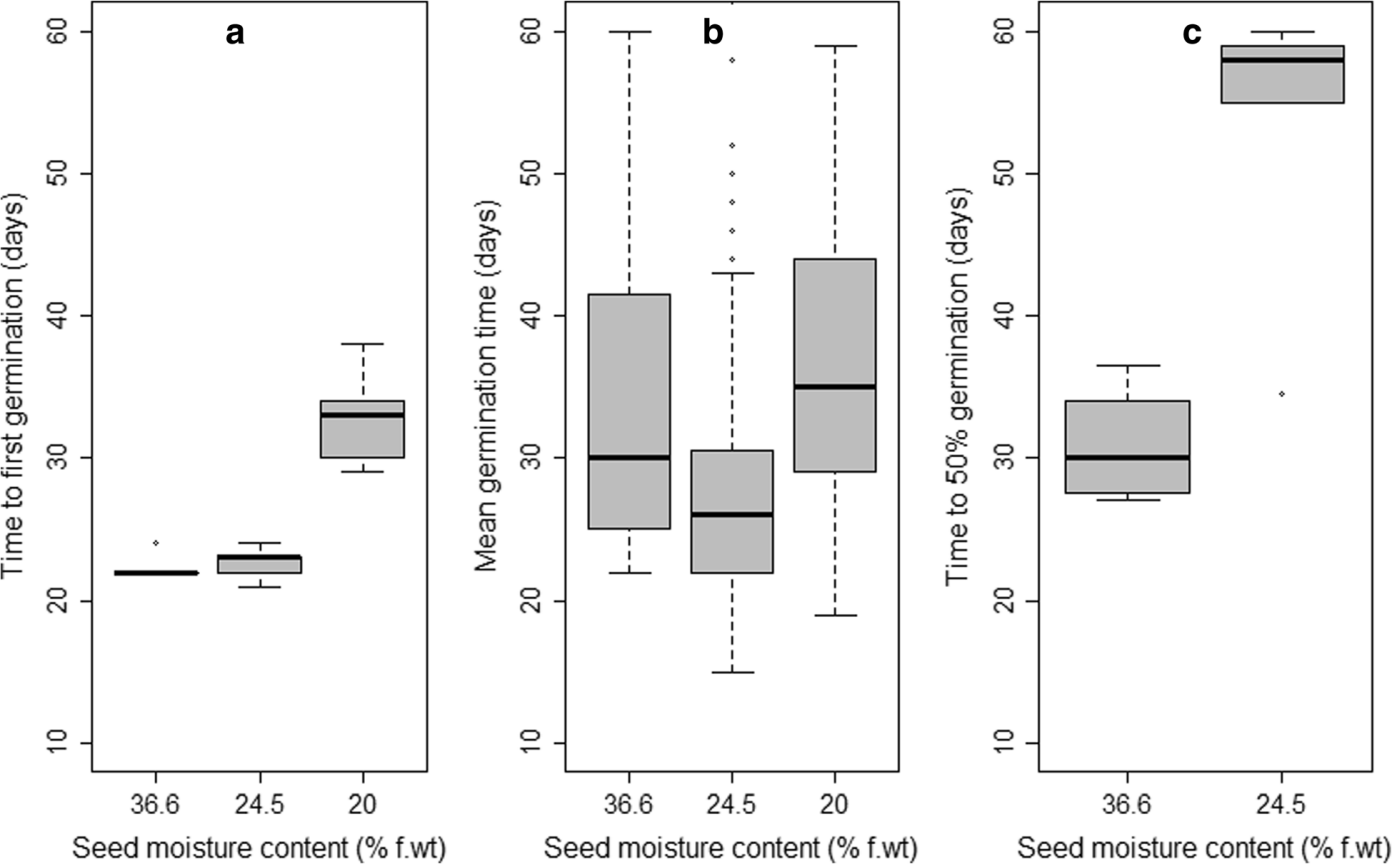
Effect of seed dehydration on the germination rate. **a** Time to first germination. **b** Mean germination time. **c** Time to 50% germination in *S. dulcificum*. In the boxplots, the ends of the box represent the 25th and 75th percentiles; the bars inside the box represent the median, and the ends of the whiskers represent the minimum and maximum values in absence of outliers (small circles below or above the whisker)

The effect of dehydration was very significant (*P* <  0.01) on TFG and highly significant (*P* <  0.001) on MGT and T_50_. Indeed, first germination occurred in average 22.4 ± 0.55 and 22.6 ± 2.47 days after sowing, respectively in fresh seeds and in seed dehydrated to 24.5%. (Fig. 4a, Additional file 3). Seed dehydrated to 20% m. c. took more time to germinate than fresh seeds or seeds dehydrated to 24.5% (Fig. 4b, Additional file 4). More importantly, the germination is by far more homogenous in fresh than in dehydrated seeds (Fig. 4c, Additional file 5). The time to reach 50% germination (T_50_) in dehydrated seeds (53.5 ± 4.82 days) was nearly the double of that of fresh seeds (31 ± 1.85 days).

Model simplification indicated no significant interaction effect between storage temperature and duration on germination rate (*P* > 0.05). The time to first germination (TFG) was not significantly affected by the storage temperature but was negatively affected by the storage duration (Table 2). Both the storage temperature and the storage duration significantly affected MGT. Germination was quicker in seeds stored at relatively higher temperature (10 °C and 25 °C) and for a shorter duration (maximum 7 days). The effect of storage temperature on the time to 50% germination (T_50_) was not significant, while in contrast the storage duration had a highly significant and negative effect on T_50_ (*P* < 0.0001) (Table 2).

**Table 2:** Generalized linear models analysis of the effect of the storage environment on the germination rate in *S. dulcificum*

Factor	Treatment	TFG	MGT	T_50_
Storage duration	3 days	21.66 ± 0.49^a^	34.04 ± 0.75^a^	29.6 ± 0.85^a^
	7 days	22.66 ± 0.98^a^	33.4 ± 0.78^a^	37.4 ± 2.04^a^
	14 days	27.40 ± 1.20^b^	38.57 ± 1.2^b^	50.8 ± 2.56^b^
	21 days	26.40 ± 0.74^b^	36.13 ± 2.39^b^	NA
	***P*** **-Value**	**0.007** ^**^	**0.0009** ^***^	**< 0.0001** ^***^
Storage temperature	25 °C	23.9 ± 0.82^a^	34.34 ± 0.69^a^	33.45 ± 2.83^a^
	10 °C	21.2 ± 0.38^a^	33.56 ± 0.80^a^	39.3 ± 1.42^a^
	4 °C	24.4 ± 1.24^a^	36.91 ± 1.37^b^	NA
	***P*** **-Value**	**0.27** ^**ns**^	**0.008** ^**^	**0.94** ^**ns**^

Values are mean ± s.e. and values within columns followed by different letters are significantly different (Tukey HSD test). *n* = 15 replicates (each replicate having 5–20 seeds) for storage duration and 20 replicates (each having 2–20 seeds) for storage temperature. Notes: TFG: time to first germination; MGT: mean germination time; T50: time to 50% germination. NA: not available (germination percentage less than 50% in the treatment); Values in Bold are probabilities; ns: not significant; ^**^: *P* < 0.01; ^***^: *P* < 0.001

### Effect of dehydration and storage temperature and duration on seedling growth in *S. dulcificum*

Sixty days after germination, the stem diameter at ground level, the height and the number of leaves were significantly lower in seedlings produced by fresh seeds (36.6% m. c.) compared to those resulting from dehydrated seeds (*P* < 0.001) (Table 3). However, no significant difference was observed in growth parameters (*P* > 0.05) between seedlings produced with seeds dehydrated to 24.50% m.c. and seedling produced with seeds dehydrated to 20% m.c.

**Table 3:** Seedling growth parameters following dehydration level and seed storage environment

Factor	Treatment	Diameter (mm)	Height (cm)	Leaf production
Dehydration	36.6%	01.27 ± 0.03^a^	02.66 ± 0.16^a^	01.95 ± 0.17^a^
	24.5%	01.51 ± 0.04^b^	03.96 ± 0.23^b^	03.01 ± 0.28^b^
	20%	01.46 ± 0.06^b^	03.98 ± 0.27^b^	03.09 ± 0.16^b^
	***P*** **-Value**	**0.002** ^**^	**< 0.0001** ^***^	**< 0.0001** ^***^
Storage duration	3 days	01.19 ± 0.01^a^	03.94 ± 0.01^a^	03.08 ± 0.85^a^
	7 days	01.23 ± 0.02^a^	03.67 ± 0.02^a^	03.13 ± 2.04^a^
	14 days	01.19 ± 0.02^a^	03.61 ± 0.02^a^	03.54 ± 2.56^a^
	21 days	01.20 ± 0.02^a^	04.00 ± 2.02^a^	03.72 ± 2.56^a^
	***P*** **-Value**	**0.65** ^**ns**^	**0.07** ^**ns**^	**0.32** ^**ns**^
Storage temperature	25 °C	01.25 ± 0.02^a^	03.88 ± 0.07^a^	03.25 ± 0.07^a^
	10 °C	01.20 ± 0.01^a^	03.87 ± 0.07^a^	03.17 ± 0.07^a^
	4 °C	01.07 ± 0.02^b^	03.30 ± 0.13^b^	02.88 ± 0.12^a^
	***P*** **-Value**	**< 0.0001** ^***^	**0.0002** ^***^	**0.22** ^**ns**^

Values are mean ± s.e. and values within columns followed by different letters are significantly different (SNK test for diameter and height and Tukey HSD test for leaf production). *n* = 5 replicates (each having 5–19 seedlings) for dehydration, 15 replicates (each having 1–18 seedlings) for storage duration and 20 replicates (each having 1–18 seedlings) for storage temperature. Note: Values in bold are probabilities; ns: not significant; ^**^: *P* < 0.01; ^***^ *P* < 0.001

There was no interaction effect between the storage duration and the storage temperature on the seedling diameter, height and number of leaf (P > 0.05). Similarly, the storage duration did not affect the seedling growth (P > 0.05). Only, the effect of the storage temperature was significant on seedling diameter and height. Overall, seedling grew poorly in seed lots stored at lower temperature. The number of leaf produced was not significantly different, regardless of the storage conditions of the seeds (Table 3).

## Discussion

Fruit colour change in *S. dulcificum* is followed by an improvement of the seed vigour. This improvement was observed from the yellowish stage and was more prominent when the fruit is fully ripe (red colour). Parameters such as germination percentages, time to first germination, and mean time to germination presented the best values for red fruits. These findings indicated that the fruit colour is a reliable indicator of seeds maturation in *S. dulcificum*, as also observed in other species. For instance, a passage from a yellow appearance to a brown appearance indicated seed maturation in *Vitis vinifera* L. cv Shiraz [29] and is illustrated through an increase in the germination performance. Likewise, Manonmani and Vanangamudi [30] highlighted that the passage of the fruit of *Santalum album* from reddish brown to black indicated seed maturation and that seeds from black fruits exhibited higher germination than seeds from fruit with any other colours. It is worth noticing that in *S. dulcificum*, the fruit colour passage to fully red occurred at 70–74 days after the flower anthesis [44]. This long post-anthesis period ensured a good maturation of seeds whereas the low value of standard errors illustrated that fully red fruits promote homogenous seed maturation in *S. dulcificum*.

**Fig. 5:**
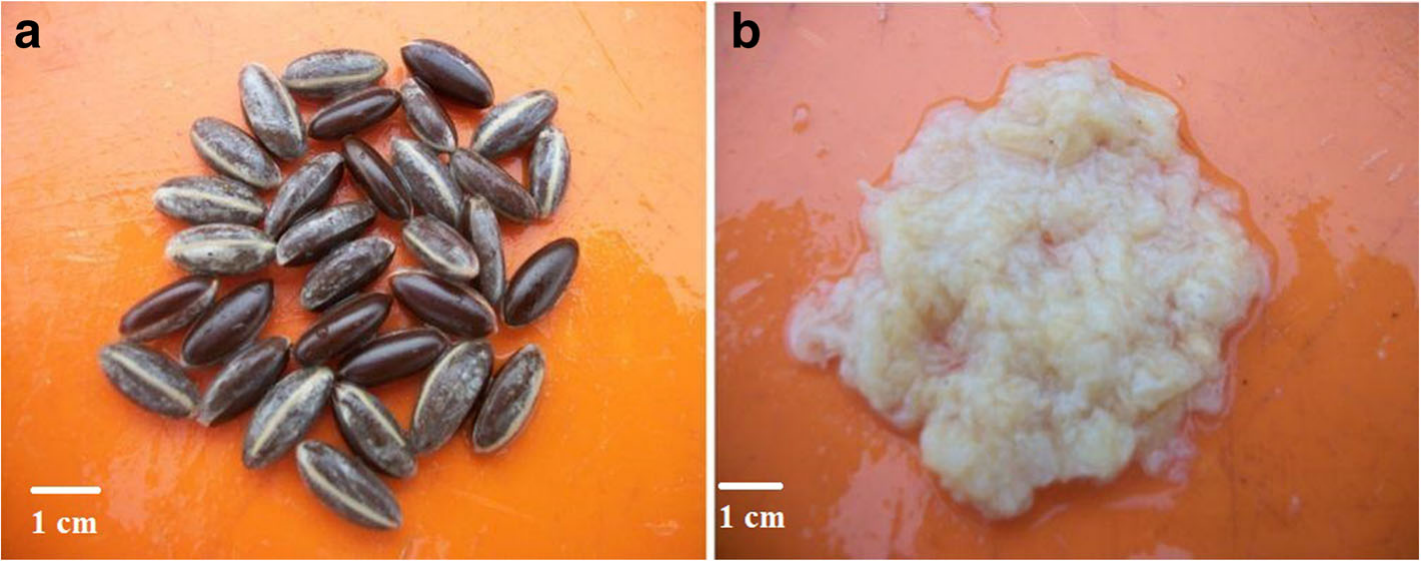
Protecting layers of *S. dulcificum* seed. **a** Shinny and smooth testa. **b** White fleshy layer covering testa

We evidenced in this study that *S. dulcificum* is truly a recalcitrant-seeded species. To date, whether *S. dulcificum* exhibited recalcitrance was only anecdotal. In their simplified protocol for seed storage determination, Hong and Ellis [3] reported that seeds of recalcitrant species were often harvested at > 30% moisture content and rarely survived to desiccation below 12–10% moisture content. In this study, the initial moisture content of *S. dulcificum* (36.6%) reflects recalcitrance behaviour. This recalcitrance behaviour was also supported by the fact that the seeds of *S. dulcificum* did not survive below 10% moisture content (e.g. 9.5 and 5.1%). In addition, based on the SCR-SM model we observed that the probability of desiccation-sensitivity [P(D-S)] of the species was consistently higher than 0.5 which is the threshold value between orthodox and recalcitrant species. According to Daws et al. [19], recalcitrant species were reported to have a P(D-S) value higher than 0.5. The SCR-SM model and the experimental approach thus converged to similar conclusions. Such a combination of approaches was also previously used by Joshi et al. [20] to clarify the intermediate storage behaviour in *Mesua ferrea* L. Moreover, the probability range of desiccation-sensitivity in *S. dulcificum* [0.76; 0.87] was comparable to those observed in other Sapotaceae species [19] such as *Vitellaria paradoxa* C.F.Gaertn [P(D-S) = 0.91] and *Pouteria sapota* (Jacq.) H.E.Moore & Stearn [P(D-S) = 0.73], supporting the hypothesis of high recalcitrance exhibition in the Sapotaceae family. Moreover, recalcitrant seeds were reported to be covered by fleshy or juicy arriloid layers and an impermeable testa [31]. This supports our observation in *S. dulcificum* in which a white flesh covers the shiny testa (Fig. 5a and b).

According to Huang et al. [32], seed moisture content and storage temperature were very determinant for the maintenance of seed viability and vigour. In addition, we found that the storage duration is another important factor. Particularly for *S. dulcificum,* storage beyond 3 weeks was detrimental for both seed viability and germination percentage [33]. The drastic viability loss observed in *S. dulcificum* following dehydration below 20% moisture content was also previously reported in other species including *Vitellaria paradoxa* C.F.Gaertn, *Cordyla pinnata* (Lepr. ex A.Rich.) Milne-Redh. and *Saba senegalensis (*A.DC.) Pichon, all of them classified as recalcitrant-seeded.

Though *S. dulcificum* exhibited a certain tolerance to short term storage (maximum 7 days) at low temperature (e.g. 10 °C or 4 °C), highest viability and germination were obtained with seeds stored at a temperature close to ambient conditions (25 °C). This is in line with indications that 10 °C and above are the optimum storage temperature for seeds of tropical native species [3, 34].

The effect of dehydration and storage on seed vigour greatly varied from one species to the other and particularly within recalcitrant species. For instance, while dehydration decreased the mean time to germinate in *Podocarpus henkelii* Stapf ex Dallim. & B.D.Jacks and in *Trichilia emetica* Vahl, it rather increased it in *Trichilia dregeana* Sond. [35]. In the case of *S. dulcificum*, dehydration decreased the seed vigour by increasing the time to first germination and especially the time to 50% germination. Seed vigour was drastically reduced by the storage temperature below 10 °C and by a storage period exceeding 2 weeks. Seed viability and germination rate then respond in a similar way to dehydration and storage environment in *S. dulcificum*.

Most studies on seed dehydration and storage in plant species and particularly in recalcitrant-seeded species [36–38] narrowed their conclusions to seed viability and germination and sometimes on seed vigour. They often overlook how seed dehydration affects subsequent seedlings performance despite the fact that seedling vigour is determinant for the survival, the functional and the productive traits of any plant species [39]. In this study, seedlings from dehydrated seeds were more vigorous than those produced by fresh seeds. This observation matches the findings by Omran et al. [40] in *Casuarina cunninghamiana* Miq. and *C. glauca* Sieber ex Spreng. but contradicts the observation by Michalak et al. [4] in *Pyrus communis* auct. Iber. where dehydrated seeds produced slow growth seedlings. Furthermore, seedling growth is known to be affected by numerous factors including for instance the gibberellin and abscisic acid ratio [41] which also can be affected by water stress and the storage duration. While gibberellin improved seedling growth, abscisic acid rather showed an inhibitory function [42]. Most experiments related to those growth regulators (e.g. function, regulators content, side effect) in plant species often used orthodox species as model [43]. Consequently, the functioning pattern of those growth regulators remained elusive in other plant groups, particularly in recalcitrant-seeded species. In *S. dulcificum,* we can speculate that seed dehydration might have increased the ratio of growth promoting hormones over growth inhibitors, resulting in bigger seedlings.

## Conclusion

This study provides for the first time data that established evidence of recalcitrance in *Synsepalum dulcificum.* Results also indicated that in one hand dehydration and low storage temperature decreased seed vigour in the species and in the other hand, seed dehydration improved seedling performance, but storage at low temperature inhibited seedling growth. Based on these findings, cryopreservation should be considered for the long-term storage of *S. dulcificum* seeds.

## Methods

We first identify the adequate seed maturity stage to be used for the experiment by comparing germination performance in fruits at different maturity stage i.e. fruits of different colours. Three fruit maturity stages including fully green fruits, yellowish, and fully red fruits, and corresponding respectively to the fruit development stages at 35–40 days, 60–65 days and 70–74 days after the flower bloom (Tchokponhoué et al. [44]) were compared. For each stage, five replications of 20 seeds each were used for the germination test. The best performing of these three fruit colours was considered as the suitable fruit development stage that ensured a sufficient seed maturation, and only seeds from this fruit colour group were considered to study the seed storage behaviour.

To elucidate the seed storage behaviour in *S. dulcificum*, we combined the experimental protocol proposed by Hong and Ellis [3] and the probabilistic prediction model proposed by Daws et al. [19]. To set the influence of dehydration and storage temperature and duration on seed vigour and seedling performance, the germination rate and seedlings vigour were compared among tested factors.

### Seed collection

Seeds used in this study were collected in the localities of Abomey-Calavi (6°25′N; 2°20′E), Klouekanmey (07°3′N; 01°49′E), Sèhouè (6°92′N; 2°27′E), and Sey (06°45′N; 02°06′E), all located in the Guinean phytogeographical area of Benin. Mature and ripe (red) fruits of *S. dulcificum* were hand-harvested from one single mother tree per locality in the localities of Klouekanmey, Sèhouè, and Sey whereas in Abomey-Calavi fruits were harvested from three different early fruiting juveniles. The three trees harvested in Abomey-Calavi were distant 10 m from each. Each lot of fruits was kept in a separate labelled white polyethylene bag and transported to the Laboratory of Genetics, Horticulture and Seed Science, University of Abomey-Calavi, Benin Republic. For each lot, seeds were extracted by gently removing the red exocarp and the white pulp from the ripe fruits. The seeds were then rinsed with tap water, wiped with a clean towel. The experiment was initiated automatically, within 8 h after fruits were harvested. Seeds collected in Sèhouè were used in the whole study (for both experimental approach and SCR-SM model) whereas those from other localities were only involved in the probabilistic prediction model (SCR-SM model).

### Seed storage behaviour: experimental approach

Only the seeds from Sèhouè were used for the experimental approach.

### Seed mass and moisture content

Four replicates of 100 fresh seeds each were weighed to the nearest 0.01 g using a digital balance (OHAUS, model: PA512, Item number: 80251571, Port Melbourne, Australia) to determine seed mass. To determine the seed moisture content at shedding, we oven (NÜVE, model: FN 055, Ankara, Turkey)-dried five samples of 20 fresh seeds each at 120 °C to a constant mass. The initial moisture content was calculated on fresh weight (f. wt) basis.

### Effect of seed dehydration on seed viability, germination rate and seedling growth

We constituted four lots of 150 fresh seeds each. Each lot was initially weighed to the nearest 0.001 g with a digital balance. Each seed lot was then air-dried at ambient laboratory conditions temperature (25–27 °C, 70% RH) to the desired moisture content (24.5, 20, 9.5 and 5.1%). The desired moisture content of each sample was monitored based on the eq. (1) [3].

1 $$ \mathrm{Weight}\kern0.3em \mathrm{of}\kern0.3em \mathrm{seedlot}\kern0.1em \left(\mathrm{g}\right)\kern0.3em \mathrm{at}\kern0.3em \mathrm{MCd}\%=\left[\left(100-\mathrm{MCi}\%\right)/\left(100-\mathrm{MCd}\%\right)\right]\times \mathrm{ISW}\left(\mathrm{g}\right) $$

with MCd being the desired moisture content, MCi the initial water content and ISW the initial seed lot weight.

When seed lots reached the target moisture content, we compared the viability, the germination percentage and rate as well as the subsequent seedling growth among dehydrated seed lots and fresh seed lot (seeds at initial m.c.) in an experimental design that is further described.

### Effect of low temperature storage on seed viability, germination percentage and rate, and seedling growth

We randomly assigned four storage temperatures (25 °C, 10 °C, 4 °C and 0 °C) and five storage duration (3, 7, 14, 21, and 28 days) to lots of 100 seeds each. Each lot of seed was stored hermetically in a small closed white plastic container. After the experimental storage, the viability, the germination percentage and rate and the subsequent seedlings growth were evaluated in each lot in an experimental design that is further described.

### Experimental design for seed viability, germination percentage and rate and seedling growth testing

We first used a simple design to evaluate the effect of seed moisture content on seed viability, seed germination percentage and rate and seedling growth in *S. dulcificum.* Five seed moisture content treatments (initial m.c., 24.5% m.c., 20% m.c., 9.5% m.c., and 5.1% m.c) were compared. Each moisture content treatment was replicated five times and each replicate contained 20 seeds. Further, a completely randomized factorial design was used to evaluate the effect of storage temperature and duration. There were 20 treatments in total, resulting from the factorial combination of the four storage temperatures and the five storage durations. Each treatment was also replicated five times and each replicate contained 20 seeds. Seeds were sown horizontally in all treatments, in a 0.75 l polystyrene nursery bag filled with soil collected at 0–10 cm horizon depth (and sterilized by heating for reducing microbial contamination) saturated to its field capacity through regular watering (twice a day) to maintain adequate soil moisture.

### Data collection

Germination was recorded daily for 60 days from the sowing date. In *S. dulcificum*, seeds were deemed germinated when their radicle protruded and is accompanied by a complete metabolic reactivation leading to the seedling being visible out of soil. At the 60^th^ day after sowing, we performed an imbibed seed crush test (a reliable alternative to the tetrazolium staining viability test) on non-germinated seeds in all treatments to separate viable seeds (seeds still alive) from dead ones [45]. In the case of *S. dulcificum*, following the imbibed seed crush test the embryos of dead seeds were yellowish or brown, and collapsed after a slight pressure and had a decay smell while those of viable seeds were greenish or white and firm when pressed. Seedlings growth (diameter, height and leaves production) was monitored in germinated seeds at the 60^th^ day after germination.

### Seed storage behaviour: SCR–SM based probabilistic model

Four seed provenances were used: Abomey-Calavi, Klouekanmey, Sèhouè, and Sey. For each provenance, we generated the probability of recalcitrance [P(D-S)] as per Daws et al. [19] following the eq. (2).

2 $$ \mathrm{P}\left(\mathrm{D}-\mathrm{S}\right)=\left({\mathrm{e}}^{3\cdot 269-9\cdot 997\mathrm{a}+2\cdot 156\mathrm{b}}\right)/\left(1+{\mathrm{e}}^{3\cdot 269-9\cdot 997\mathrm{a}+2\cdot 156\mathrm{b}}\right) $$

With P(D-S) being the probability for seeds of the species to be recalcitrant, **a** being the seed coat ratio (SCR) and **b** being the log_10_ [seed dry mass (g)]. To determine parameters **a** and **b** we used 10 seeds (more than 8 seeds recommended by Daws et al. [19]) per mother tree per provenance. To determine the seed coat ratio, we first separated seed coat and albumen of all seeds and then oven-dried each component to a constant mass at 120 °C.

### Data analysis

The viability (Vp) and the germination (Gp) percentages were calculated for each treatment whereas the germination rate was assessed using the time to first germination (TFG) [46], the mean germination time (MGT) [47] and the time for 50% germination (T_50_) [48]. This latter parameter was determined only for treatments in which at least 50% germination was recorded. Vp, Gp and MGT were respectively computed as:

3 $$ {\mathrm{V}}_{\mathrm{p}}=\left({\mathrm{N}}_{\mathrm{vp}}+{\mathrm{N}}_{\mathrm{gp}}\right)\times 100/\mathrm{N} $$

4 $$ {\mathrm{G}}_{\mathrm{p}}=\left(\mathrm{N}\times 100\right)/\mathrm{N} $$

5 $$ \mathrm{MGT}={\sum}_{\mathrm{i}=1}^{\mathrm{k}}\left({\mathrm{n}}_{\mathrm{gpi}}\times {\mathrm{t}}_{\mathrm{i}}\right)/{\sum}_{\mathrm{i}=1}^{\mathrm{k}}{n}_{gpi} $$

with Nvp being the number of non-germinated seeds which were still alive in a treatment p after the imbibed seed crush test, Ngp the number of seeds that germinated in a treatment p, N the total number of seeds sown in the treatment (here 100 seeds for each treatment), t_i_ is the duration since germination experiment started up to date i, and n_gpi_ the total number of seeds in treatment p that have germinated at date i. The time to first germination (TFG) was assessed as the day when the first seed germinated in a replicate and T_50_ as the day when 50% of seeds have germinated in each replicate.

To assess the effect of dehydration and the interaction effect between the storage temperature and the storage duration on the viability and the germination percentages we used a generalized linear model (glm) with a binomial error structure or quasi-binomial error structure (to account for overdispersion) where necessary. To evaluate the effect of the same parameters on the germination rate (TFG, MGT, and T_50_), we used a glm with a Poisson error structure or a negative binomial error structure (to account for overdispersion) where necessary. For glm models we used Tukey HSD test to separate means when significant difference was observed. We used an analysis of variance depicted by contrast analysis or SNK test to highlight effect of treatments on diameter and height growth in 2 months old seedlings. To analyse the effect of treatments on leaf production in 2 months old seedlings we used a glm with a quasi-poisson error structure. We used a binomial test to check whether the seed origin and the mother plant growth stage induce any significant difference in the probability of recalcitrance in the species**.** All analyses were performed in R [49] (version 3.3.2) through RStudio [50] (version 1.044) using “agricolae”, “gdata”, “MASS” “multcomp” packages in addition to basic ones. Results were presented as mean ± standard error (s.e.).

## Additional files

Additional file 1: Dataset supporting analyses of the effect of dehydration on seed viability and germination. (XLS 41 kb)

Additional file 2: Dataset supporting analyses of the effect of the storage environment on seed viability and germination. (XLSX 17 kb)

Additional file 3: Dataset supporting analyses of the effect of dehydration on the time to first germination. Viability and germination. (XLSX 15 kb)

Additional file 4: Dataset supporting analyses of the effect of dehydration on the mean germination time. (XLSX 16 kb)

Additional file 5: Dataset supporting analyses of the effect of dehydration on the time to 50% germination. (XLSX 14 kb)

## Abbreviations

Gp: Germination percentage
MGT: Mean germination time
P(D-S): Probability for dessication-sensitivity
SCR-SM: Seed-coat ratio–Seed dry mass
T50: Time to 50% germination
TFG: Time to first germination
Vp: Viability percentage
